# Feasibility and Effectiveness of an Automated Bilingual Text Message Intervention for Weight Loss: Pilot Study

**DOI:** 10.2196/resprot.2789

**Published:** 2013-11-06

**Authors:** Julia K Kolodziejczyk, Gregory J Norman, Angelica Barrera-Ng, Lindsay Dillon, Simon Marshall, Elva Arredondo, Cheryl L Rock, Fred Raab, William G Griswold, Mark Sullivan, Kevin Patrick

**Affiliations:** ^1^Center for Wireless & Population Health Systems (CWPHS), Qualcomm Institute/Calit2University of California, San DiegoLa Jolla, CAUnited States; ^2^Department of Family & Preventive MedicineUniversity of California, San DiegoLa Jolla, CAUnited States; ^3^Graduate School of Public HealthSan Diego State UniversitySan Diego, CAUnited States; ^4^Computer Science & EngineeringUniversity of California, San DiegoLa Jolla, CAUnited States

**Keywords:** physical activity, diet, obesity, health behavior, Hispanic Americans, weight loss, cellular phone, text messaging

## Abstract

**Background:**

Little is known about the feasibility and acceptability of tailored text message based weight loss programs for English and Spanish-language speakers.

**Objective:**

This pilot study evaluated the feasibility, acceptability, and estimated impact of a tailored text message based weight loss program for English and Spanish-language speakers. The purpose of this pilot study was to inform the development of a full-scale randomized trial.

**Methods:**

There were 20 overweight or obese participants (mean age 40.10, SD 8.05; 8/20, 40% male; 9/20, 45% Spanish-speakers) that were recruited in San Diego, California, from March to May 2011 and evaluated in a one-group pre/post clinical trial. For 8 weeks, participants received and responded to 3-5 text messages daily sent from a fully automated text messaging system. They also received printed weight loss materials and brief 10-15 minute weekly counseling calls. To estimate the impact of the program, the primary outcome was weight (kg) measured during face-to-face measurement visits by trained research staff. Pre and post differences in weight were analyzed with a one-way repeated measures analysis of variance. Differences by language preference at both time points were analyzed with *t* tests. Body mass index and weight management behaviors also were examined. Feasibility and acceptability were determined by recruitment success, adherence (ie, percentage of replies to interactive text messages and attrition), and participant satisfaction.

**Results:**

Participants who completed the final assessment (N*=*18) decreased body weight by 1.85 kg (*F*_1,17_=10.80, *P*=.004, CI_∆_ 0.66-3.03, η^2^=0.39). At both time points, there were no differences in weight by language preference. Participants responded to 88.04% (986/1120) of interactive text messages, attrition rate was 10% (2/20), and 94% (19/20) of participants reported satisfaction with the program.

**Conclusions:**

This fully automated text message based weight program was feasible with English and Spanish-speakers and may have promoted modest weight loss over an 8-week period.

**Trial Registration:**

Clinicaltrials.gov NCT01171586; http://clinicaltrials.gov/ct2/show/NCT01171586 (Archived by WebCite at http://www.webcitation.org/6Ksr6dl7n).

## Introduction

Text message based programs to promote behavior change are a rapidly growing area of research. This inexpensive, instantaneous, two-way communication of brief written messages via a mobile phone has many capabilities that may be useful for promoting weight loss. For example, texting features can support important constructs in behavior change theories such as cues to action, reinforcement, goal setting, goal reminders, and feedback. Text messages can be used as a stand-alone program [1-4] or can be integrated easily with other wireless or networked technologies [5,6]. Studies have demonstrated that text messages promote improved diet [5], increased physical activity (PA) [4,7-10], behavioral strategies like self-monitoring [3], and weight loss [1,2,6,11,12]. However, more research is needed regarding long-term efficacy and best practices of these programs, in particular in diverse populations.

This pilot study evaluated feasibility and acceptability of a tailored text message based weight loss program for English and Spanish-language speakers and enabled an estimate as to its impact on weight status. The purpose of this pilot study was to inform the development of a full-scale randomized trial.

## Methods

### Unblinded One-Group Pre/Post Design

We used an unblinded one-group pre/post design. The Institutional Review Board of University of California, San Diego (UCSD) approved this study. This manuscript is in accordance with the CONSORT-EHEALTH checklist [13] and is a registered trial (NCT01171586).

### Recruitment

Participants were recruited in San Diego, California, from March to May 2011 via newspapers, flyers, online announcements, and participant recommendations. The first 20 individuals who met the eligibility criteria were enrolled, with a goal of 40% (8/20) male and 50% (10/20) self-identified Spanish-speaking.  Bilingual speakers choose language-message preference.

Eligible individuals were 21-60 years of age, had a body mass index (BMI) of 27.0-39.9, had a cellphone capable of sending and receiving text messages, were current users of texting or willing/able to learn, and could communicate in English or Spanish. Participants were excluded if they could not engage in moderate intensity PA, were pregnant or intended to become pregnant during the study, had a history of substance abuse or psychiatric disorders that would impair compliance, were using weight-altering medications, or were enrolled in another weight loss program. At baseline, potential participants were screened for inclusion and exclusion criteria and underwent written informed consent. Participants were compensated $75 for participation and $10 for a text message plan.

### Intervention

Social cognitive theory [14], control theory [15], and ecological theory [16] informed the intervention. It integrated these theoretical approaches with evidence-based behavioral strategies for improving diet and PA. Strategies include self-monitoring, intention formation, goal setting, goal review, feedback on performance, self-efficacy, benefits, barriers, problem-solving, social support, and tailoring.

The 8-week intervention included: (1) 3-5 automatically scheduled and tailored text messages per day. Message content focused on diet and PA weight management behaviors and strategies; (2) a printed weight loss binder organized by weekly weight management topics such as portion control, increasing PA, reducing sedentary behavior, and self-monitoring; and (3) brief weekly 10-15 minute counseling calls to provide encouragement and reinforcement. A database was developed of more than 3000 text messages. The research group translated and culturally tailored the messages to Spanish-speakers to ensure linguistic and cultural equivalence. Approximately one-quarter of messages requested a reply, with the balance providing tips, suggestions, and positive reinforcement or encouragement for improved behaviors. The following shows sample messages sent and received from ConTxt (San Diego, CA 2011): (1) ConTxt–What is your weight today?, (2) Participant–220, (3) ConTxt–Congratulations! You have lost 5 lbs since starting ConTxt, (4) ConTxt–Here’s a healthy tip, put your pedometer on your nightstand so you can remember to put it on in the morning, (5) ConTxt–Work on your goal of reducing portion sizes this week by buying single serving pre-packaged snacks, and (6) ConTxt–Thank you. Your response has been recorded. A total of 1500 rules were added to control what message was sent based on the weekly behavioral strategy, day of the week, and time of day, as well as other parameters such as self-reported weight management behaviors and pedometer step count. A baseline dietary assessment of weight management behaviors was conducted using the Weight Behavior Inventory (WBI) to identify unique diet and PA behavior challenges for each participant contributing to high-energy intake and low-energy expenditure. A computerized expert system processed these data to create individualized goals based on predetermined logic rules. Goals were presented to the user via text message to serve as prompts for behavioral improvements. The system is designed so participants who show rapid and sustained progress can advance through content, while those experiencing difficulties can receive additional tips and suggestions.

### Measures

Outcomes were measured at baseline and 8 weeks by trained research staff at UCSD research offices during face-to-face visits. The primary outcome was weight (kg) measured using a calibrated scale. Secondary measures included BMI calculated as kg/m^2^ and weight management behaviors associated with weight loss measured with the 35-item WBI (validation study under review, Kolodziejczyk et al 2013). The WBI was adapted from the validated Eating Behavior Inventory [17,18]. Each behavior on the WBI is rated on a five-point scale. Total scores are averaged and can range from 1-5. Sample items include “I keep one or two raw vegetables available for snacks” and “I decide ahead of time what I will eat for meals and snacks.”

Feasibility and acceptability were measured by recruitment success, adherence, and participant satisfaction. Recruitment was deemed successful if we achieved our enrollment goal of 20 participants in two months. Adherence was measured by percentage of replies to interactive text messages (ie, text messages requesting a reply) and attrition rate. Satisfaction was measured using a Likert scale that asked about level of satisfaction with the program, as well as program components. In addition, we asked open-ended questions about elements of the program such as what they liked the least and best.

### Statistical Analysis

Pre and post differences between weight, BMI, and WBI scores were analyzed with one-way repeated measures analysis of variance. WBI score differences between gender and language preference at each time point were analyzed with independent sample *t* tests. Analyses used an alpha level <.05 and were conducted using SPSS Statistics 17.0 (SPSS Inc, Chicago, Illinois).

## Results

### Participants

A total of 18 out of the 20 participants completed all measures (ie, two participants completed the program but did not show for their final assessment). On average, the sample was obese and had approximately equal percentages of participants across demographic categories. Table 1 displays participant demographics.

**Table 1:** Demographic characteristics of the ConTxt pilot participants (N=20).

Demographic variables		Overall sample
Age at study entry in years, mean (SD)		40.10 (8.05)
BMI (kg/m^2^), mean (SD)		33.67 (4.00)
Female, n (%)		12 (60)
**Education, n (%)**		
	Trade or technical school	6 (30)
	Some college	2 (10)
	College graduate	3 (15)
	Graduate degree	7 (35)
	“Prefer not to answer”	2 (10)
Married, n (%)		11 (55)
**Race/ethnicity, n (%)** ^a^		
	Hispanic	15 (75)
	White non-Hispanic	13 (65)
	Asian	2 (10)
	“Prefer not to answer”	4 (20)
**Language preference, n (%)**		
	English	11 (55)
	Spanish	9 (45)
**Monthly income, n (%)**		
	$1,000-1,999	5 (25)
	$2,000-3,999	5 (25)
	$4,000-5,999	6 (30)
	≥ $6,000	3 (15)
“Don’t know/prefer not to answer”		1 (5)

^a^More than one race category may apply

### Participants’ Body Weight

Participants decreased body weight by 1.85 kg (*F*_1,17_=10.80, *P*=.004, CI_∆_ 0.66-3.03, η^2^=0.39), decreased BMI by 0.70 kg/m^2^ (*F*_1,17_=13.21, *P*=.002, CI_∆_ 0.29-1.11, η^2^=0.44), and increased WBI scores by 0.56 points (*F*_1,17_=14.51, *P*=.001, CI_∆_ 0.25-0.87, η^2^=0.46) (Table 2). At baseline, there were no differences in WBI scores by gender (*t*_18_=0.71, *P*=.48, CI_Δ_ −0.31 to 0.62). There were no baseline differences by language preference and weight (*t*_18_=0.14, *P*=.89, CI_Δ_ −15.44 to 17.60), BMI (*t*_18_= −0.51, *P*=.61, CI_Δ_ −4.80 to 2.91), or WBI scores (*t*_18_=1.01, *P*=.33, CI_Δ_ −0.23 to 0.67). At 8 weeks, there were no differences in WBI scores by gender (*t*_16_=0.81, *P*=.43, CI_Δ_ −0.54 to 0.24), but participants preferring Spanish language had higher WBI scores (mean 2.87, SD 0.33) than English-preference participants (mean 2.48, SD 0.32; *t*_16_=−2.60, *P*=.02, CI_Δ_ −0.72 to −0.07). There were no differences at 8 weeks by language preference and weight (*t*_16_=0.07, *P*=.95, CI_Δ_ −19.16 to 20.47) or BMI (*t*_16_=−0.36, *P*=.73, CI_Δ_ −5.34 to 3.80).

**Table 2:** Weight, BMI, and WBI from the ConTxt pilot study (San Diego, CA 2011) at baseline and 8 weeks (N=18).

Outcome	Baseline	8 weeks	% Change
Weight (kg)^a^, mean (SD)	92.96 (39.65)	91.11 (42.41)^b^	-1.99
BMI (kg/m^2^), mean (SD)	33.78 (4.16)	33.07 (4.45)^b^	-2.10
WBI score (points)	2.11 (0.49)	2.67 (0.37)^c^	26.54

^a^kg= kilograms

^b^ *P*<.01

^c^ *P*<.001

### Participant Interest

There was considerable interest in the study, as the recruitment goal of enrolling 20 participants in two months was achieved quickly after receiving 123 inquiries. Participants responded to 88.04% (986/1120) of interactive text messages, and there was a low 10% (2/20) attrition rate. Most participants (94%, 19/20) reported satisfaction with the program. Participants also reported the program helped motivate and reinforce healthier habits and choices (n=5), encouraged portion control and awareness of energy intake (n=4), and taught how to be more active (n=5). Some challenges participants reported included feelings of withdrawal after the program ended (n=7) and technical issues with their phone, which sometimes hindered message response (n=4).

## Discussion

### The Weight Loss Program

An 8-week text message based weight loss program was found to be both feasible and acceptable in terms of recruitment interest, participant adherence, and satisfaction. The program may have had positive effects on weight management behaviors and weight outcomes, although this needs to be confirmed in a study with a stronger design. These results are consistent with previous text message based weight loss studies [1,2,11]. Based on information we received from this pilot, some changes to be implemented in the full-scale trial include user-initiated messages (eg, suggestions for restaurant meals, PA), more message personalization (eg, names of social supporters, PA locations), a greater focus on participant message preference (eg, participants will be able to set text message preferences through the use of a like/unlike system), inclusion of “milestone” and “competitive” messages based on weight and pedometer step count (eg, when a participant reaches a certain milestone, such as five pounds lost, he or she will receive a congratulatory message, and the system will compare the participant’s weight loss with the groups’ weight loss), and improvements to system programming to reduce technical errors (eg, a participant not receiving the correct follow-up message). In addition, based on feedback from the Spanish-language speaking participants, we made some of the Spanish materials clearer (eg, more pictures, simpler language).

### Study Limitations

Study limitations include a small sample, a short time frame, and a one-group pre/post design. Therefore, our findings are suggestive rather than conclusive. Based upon these pilot study findings a full-scale randomized controlled trial currently is underway enrolling 298 participants for a one-year intervention.

## Abbreviations

BMI: body mass index
PA: physical activity
UCSD: University of California, San Diego
WBI: Weight Behavior Inventory
